# A Perspective on Home-Based Sexual Health Care: Evidence, Access, and Future Directions

**DOI:** 10.1007/s11904-025-00724-5

**Published:** 2025-02-26

**Authors:** Cornelia J.D. (Hanneke) Goense, Ymke J. Evers, Christian J.P.A. Hoebe, Nicole H.T.M. Dukers-Muijrers

**Affiliations:** 1 Department of Sexual Health, Infectious Diseases and Environmental Health, Living Lab Public Health, Public Health Service South Limburg, P.O. Box 33, Heerlen, 6400 AA The Netherlands; 2 Department of Health Promotion, Care and Public Health Research Institute (CAPHRI), Maastricht University, Maastricht, Netherlands; 3 Department of Social Medicine, Care and Public Health Research Institute (CAPHRI), Maastricht University, Maastricht, Netherlands; 4 Department of Medical Microbiology, Infectious Diseases and Infection Prevention, Care and Public Health Research Institute (CAPHRI), Maastricht University Medical Centre+ (MUMC+), Maastricht, Netherlands

**Keywords:** Home-based sexual health care, STI testing, HIV testing, Perspective, Implementation

## Abstract

**Purpose:**

This perspective explores the impact of home-based sexual health services on accessibility to STI and HIV testing for priority populations. This study evaluates home-based services as independent care options and as complementary components of traditional clinic-based care.

**Recent Findings:**

Challenges for persons to attend clinic-based sexual health care can be overcome by offering lower threshold home-based sexual health care. Implementing home-based services has successfully reached priority populations, including previously untested men who have sex with men (MSM) and individuals with a high exposure risk for sexually transmitted infections (STI) including human immunodeficiency virus (HIV), attending location-based sexual health care. A challenge in home-based services is to ensure equitable care, such as for individuals with limited access to digital resources or low health literacy.

**Summary:**

While home-based sexual health services enhance the accessibility of sexual healthcare, to ensure equitable care, research into the needs of still underserved populations and subsequent tailoring of the care offered, is needed. Continuous monitoring and evaluation of the implementation of home-based services may maximize the advantages of this promising type of care.

## Introduction

In many global and national public health strategies, priority populations are identified for intervention and resource allocation for sexually transmitted infections (STI) including human immunodeficiency virus (HIV). Individuals in priority populations can benefit from targeted sexual health care interventions to make a significant impact on both individual and public health. Individuals from priority populations may encounter barriers to accessing location-based sexual health care, including feelings of shame, anticipated stigma from healthcare providers, and logistical difficulties such as distance to clinics and transportation costs, particularly in rural areas where such clinics are scarce [1–3]. Offering testing and care outside traditional clinics can help mitigate these barriers, making sexual health care more accessible. During public health crises like the COVID-19 pandemic, home-based sexual health services ensure the continuity of care [4, 5]. Additionally, these services provide an accessible and cost-effective approach to regular STI and HIV testing, facilitating early diagnosis and treatment [6]. From an organizational perspective, implementing home-based care can alleviate the burden on clinic-based services by reducing the workload of healthcare providers and allowing resources to be redirected toward reaching vulnerable populations. This shift not only enhances service efficiency but also supports equitable access to sexual health care, location or home-based, for diverse patient groups [7–9].

Home-based sexual health care includes testing for either or both STI and HIV [10]. Furthermore, home-based sexual health care may present with diverse elements, for instance with self-sampling with laboratory testing or self-testing where the patient self assesses the results [11]. Stand-alone home-based sexual health care is widely available as paid or commercially available self-(sampling) tests to order online or buy in stores. Also, home-based care services were found to be integrated within existing location-based care. Main implementers then are health care providers of public health services, STI clinics, or general practitioners [12]. Some examples of home-based services with self-collection at home and laboratory analysis for STI testing are “I Want the Kit” [13], ‘TakeMeHome’ [14], Project Caboodle [15]!, Preventx online postal self-sampling [16], MemoDepistages [17], and a Dutch regional home-based sexual health care service “Limburg4zero” [18]. Other examples of home-based care services mostly use HIV self-testing options, where patients need to self-assess their results [19–22].

This perspective aimed to explore the evidence of home-based sexual health care regarding the accessibility of care for priority populations, and the benefits and challenges of home-based care. In addition, implementation possibilities and future developments of home-based sexual health care were explored.

### A Perspective on Home-Based Sexual Health Care

Home-based sexual health care can be viewed as “comprehensive” when it consists of a combination of the key elements of home-based testing (self-testing or self-sampling testing) for STI or HIV, additional counseling through (tailored) online sexual health information or e-health services, and referral to obtaining treatment or further needed (medical) care [10]. Key elements of the implementation of home-based care include tailoring the care service and its dissemination to the priority population (formerly also referred to as ‘key population’), including ensuring the provision of clear and accessible instructions.

Whereas in recent past, all priority populations who sought sexual health care had to attend location-based care, the option of home-based sexual health care now allows relocation of care to the home setting [23]. Most reports evaluating home-based care and its implementation were focused on men who have sex with men (MSM) [24], including previously untested MSM at risk for STI and HIV exposure. Several studies confirm the successful reach of this population. A Dutch regional home-based sexual health care service for MSM demonstrated that approximately 40% of the users never had tested before [18]. Home-based STI and HIV testing services in Asian countries have also demonstrated success in reaching previously untested MSM [19, 25]. A recent randomized controlled trial among MSM in the US found that self-testing motivated frequent HIV testing among those who had never tested before, supported by the option of eHealth (eTest) follow-up and sexual health counselling [5]. This study reported that home-based testing contributed to the diagnosis of new HIV cases [20]. Another home-based service in the US (TakeMeHome) identified new cases of STI and HIV [14].

In addition, home-based sexual health care is an acceptable alternative to location-based care for MSM who previously attended location-based care. A positive attitude and high intention to use home-based sexual health care were demonstrated among Dutch MSM who previously attended location-based care [26]. A French home-based sexual health care service (MemoDepistages) also reached out to MSM who had a previous test experience [27]. Uptake of self-sampling testing was particularly high among MSM who reported to have sex with multiple partners.

### Evidence for Advantages and Challenges

Table 1 shows an overview of the advantages and challenges of diverse forms of sexual health care, comparing location-based sexual health care, comprehensive home-based sexual health care integrated in routine (STI clinic) care, and home-based testing services offered commercially. A recent scoping review evaluated stand-alone STI tests that a person could order online across the US [28], highlighting the value of this care-for in reaching patients who experience challenges in attending location-based care. The offered care services are usually found acceptable by its users. For example, a recent qualitative study among MSM in the US (Project Caboodle!) demonstrated a highly positive attitude towards using home-based sexual health care services. Home-based testing could lower barriers to STI and HIV testing for MSM who expected or experienced challenges (e.g., stigma, transportation) in attending location-based care [3]. Participants particularly praised the discretion and usability of the test kits [15]. Providing populations with various options for STI and HIV testing, to choose from, at home or at location, enhances their autonomy and self-control over their own sexual health [25, 29]. Another example is a qualitative study (TINY vial) among MSM in the UK, demonstrating an increased sense of independence and autonomy [30]. An example of a challenge experienced by home-based care users is that blood self-collection using a finger prick in home-based care, can be a complex procedure for some patients [31, 32].

**Table 1:** Advantages and challenges of different sexual health care services

	Advantages	Challenges
**Location-based sexual health care at an STI clinic**	- Standard of care available - Availability of prevention and counseling services - Opportunity to immediately examine symptoms by a health care provider in person - Referral to treatment or direct medication provision, and partner-management services	- May not be accessible for various populations who experience barriers to location-based care - No show at the clinic
**Home-based comprehensive sexual health care offered by health care providers** (this is a combination of self-(sampling) testing, additional counseling options, treatment, and referral)	- Standard of care available - Accessible for priority populations for whom the service was designed - Tailoring of direct online counseling and advice and opportunity for chat or webcam consultation - Professional help (knowledge, skills, advice) (remotely) available regarding sexual health care	- Lost-to-follow-up - Missing opportunities for further sexual health counseling - May be less accessible for populations for which the service was not specifically designed
**Commercialized (stand-alone) home-based sexual health care** (this is paid self-(sampling) testing services)	- Accessible for large group of people - Higher return rate of (paid) self-sampling tests	- Lost-to-follow up - Standard of care unknown - Missing opportunities for further care and prevention - May not be accessible for individuals with lower financial resource

An advantage of home-based care is its reach in populations that are underserved or at highest exposure risk. An Australian surveillance study assessed HIV test uptake following the introduction of commercial (paid) HIV self-tests [33]. While the overall increase in home-based HIV testing was modest, the study indicated effective reach among priority populations, including MSM with irregular testing histories, migrants, and individuals at elevated risk for HIV exposure, who were more likely to test at home. This suggests that both care-integrated and commercially provided at-home testing may enhance the accessibility of sexual health care. However, when testing services are not administered by a health care provider, the quality and standard of care may not be assured [23]. Concerns regarding commercially provided tests primarily focus on the accuracy of self-tests and the confidentiality of the testing process, and challenges in high testing costs for the patient. These challenges are typically absent in home-based care offered by public health care [18, 33]. 

### Improving Accessibility of Care for Underserved Priority Populations

Individuals from priority populations may face barriers to accessing location-based sexual health care including stigma and logistical or other contextual challenges, particularly in rural areas. Home-based care offers an alternative that addresses these barriers by providing accessible testing and services outside traditional clinics. The specific tailoring of home-based care to underserved groups, could contribute to reduce inequity in access to healthcare. Nevertheless, a scoping review on outcomes for home-based testing by online postal service described the potential of growing inequity induced by this type of care [34]. This occurs when the care service matches the needs, preferences, and skills of certain members of priority populations while being incongruent with those of other priority populations. The scoping review suggested that home-based sexual health care may be most suitable for individuals who already had used location-based care, but possibly less accessible for more vulnerable individuals who have sexual health concerns or who were unfamiliar with location-based care.

The implementation evaluation of the Dutch “Limburg4zero” revealed that while the priority population of untested MSM was effectively reached, there was an underrepresentation of potentially vulnerable subgroups, such as MSM with less than a high school education and migrants of non-Western ethnicity, highlighting potential underlying inequities in access [18]. Continuous evaluation and enhancement of sexual healthcare accessibility for diverse underserved priority groups remains essential. A realist review revealed the importance of contextual elements herein [10]. Design of tailored communication messages, harmonized with the needs of the priority population, may enhance risk perception, self-control, and general STI and HIV knowledge [10].

To be able to tailor a service to the needs of the priority population, what is needed is an ongoing evaluation of the uptake and implementation of the service and its associated factors, including the dissemination and communication of the care service, and allowing for systematic adaptation of both the service and the implementation process. The high customizability of home-based care services should be fully utilized to optimize both the service itself for various priority populations and the implementation by health care providers [35]. For example, translating testing information to diverse languages enhances the accessibility of the service for MSM. A study in Cameroon demonstrated successful uptake of HIV self-testing when materials were available in regional languages [36]. Additional consultation options with a health care provider can be built in (for instance via phone or by webcam), as reportedly favored by MSM on pre-exposure prophylaxis (PrEP) when they would use home-based testing rather than location-based care; these MSM valued face-to-face consultations with an health care provider when testing for STI and HIV [31]. A previous RCT among male couples also suggested additional video counseling with an health care provider when STI testing at home (Project Nexus), especially when receiving positive results to improve linkage to care [37]. A global systematic review supports the addition of digital counseling options, such as by webcam, specifically when self-testing and self-assessing the results [38]. Webcam consultations can be a digital alternative to or complement in-person contact at a clinic location. However, challenges may be encountered such as organizational limitations in (information or digital) technological possibilities, the financial resources for such innovations, and a lack of process management when tailoring a health care innovation [39].

### The Role of Health Care Providers in Successful Implementation

Public health care providers at an STI clinic can implement home-based sexual health care services in close collaboration with other health care providers, such as general practitioners or hospital-based specialists, and with community-based organizations and community-members [18]. Implementing home-based sexual healthcare can speed up care provision for priority populations, it may help reduce location-based patient load, and thereby reduce waiting times overall. Home-based caremay also allow health care providers to allocate time to other critical tasks, such as attending to more vulnerable individuals including persons with low health literacy [39]. Previous studies have reported health care provider task shifting and increased work efficiency when implementing home-based sexual health care [9, 18].

A concern expressed by health care providers was the potential loss of direct patient contact in home-based care, regarding possible counseling advantages of in-person interaction. Although health care providers have generally expressed willingness to implement home-based care services, they were uncertain about patients’ follow-up on positive results and missed opportunities for prevention [9]. This was also demonstrated in a Dutch implementation of home-based sexual health care, where nurses provided detailed examples of concerns about the quality of care such as a lack of direct contact with patients, which may result in missing (non-verbal) signals and, consequently, hinder the ability to deliver appropriate care and prevention [18].

For the care organization, the long-term cost-effectiveness of implementing home-based sexual health care is considered an advantage [7, 40]. It is noted that initial implementation may require higher costs due to the expenses involved in starting process. These costs can include extra staffing, setting up collaborations with stakeholders, putting necessary systems in place, and establishing monitoring processes [41].

A qualitative evaluation study among staff of an Australian sexual health care service outside a clinic (MyCheck) highlighted the value of allowing patients to decide on where to get tested [42]. A US study concluded follow-up possibilities with eHealth services as a beneficial addition when, in this case, MSM used HIV self-testing [20]. MSM reportedly preferred home-based sexual health care as a complimentary option to location-based services [26, 31]. An American modeling study suggested that supplementing HIV self-testing with regular location-based sexual health care could contribute to reducing HIV incidence [43].

### Implementation Opportunities

An important and common concern associated with health care innovations is the risk of inadequate implementation. Also in home-based sexual health care, various elements of the intended implementation strategy (i.e., registration processes, communicational strategies, monitoring) are not always put into practice, preventing the intended advantages of the home-based service from being secured in real life care practice [44]. When implementating, it is crucial to have knowledge about the (determinants of) behavior of professionals in adopting, implementing, and continuing the intervention as intended, as well as knowledge about the various contextual factors, in order to optimize the implementation success of the home-based care innovation.

A wide number of studies described the implementation of home-based sexual health care, but only a few studies to date reported using a systematic implementation framework. The Practical, Robust, Implementation and Sustainability Model (PRISM) is one such framework, and may be utilized to guide, optimize, and evaluate the implementation process systematically [45]. A study on the implementation of HIV testing in a workplace (Test@Work) in the United Kingdom assessed that the RE-AIM framework aided in evaluating the intervention and assessed the challenges of implementing it [56]. An Australian peer-led STI screening program (Deadly Liver Mob) highlighted that RE-AIM enabled a clear description of the implementation process in a real-world setting [57].

The PRISM framework addresses key outcomes of the implementation strategy which are: the Reach of the priority population, the Effectiveness of the intervention, Adoption of the intervention, Implementation fidelity, and long-term Maintenance (in short RE-AIM), brought together with contextual domains such as external environment and sustainability infrastructure [46]. A key feature of PRISM is that it includes the validation of the role of ‘context’. The importance of contextual domains for the understanding of implementation success is also stressed in a qualitative study on the use of the Consolidated Framework for Implementation Research (CFIR), which is another frequently used implementation framework [47].

Ideally, an implementation framework should be used before and also throughout implementation, in (1) selecting framework(s) and behavioral theories, (2) establishing and maintaining stakeholder engagement, (3) defining and developing evaluation questions, (4) developing an implementation mechanistic process model or logic model (i.e., visually linking resources and outcomes), (5) selecting evaluation methods (6) determining implementation factors, (7) selecting, tailoring, or developing, implementation strategy(s), (8) specifying implementation outcomes and evaluating implementation, (9) using the framework(s) on a limited scale (pilot) to tailor implementation, and (10) reporting [48]. A challenge of the PRISM framework is its broad scope, which can complicate operationalization, and demand significant effort to assess the multiple interacting factors across diverse implementation contexts comprehensively.

### Future Directions

Future developments may be focused on the optimization of home-based sexual health care service itself and the optimization of implementation strategies. For example, improving the care service by enhancing serology sampling methods, such as easier high-volume blood self-collection (other than via finger-prick), and improving implementation by including digital developments, such as the use of artificial intelligence (AI). In recent years, advancements in home-based sexual health care have accelerated, offering more accessible and efficient solutions. Particularly since the global COVID-19 pandemic home-based sexual health care services have been increasingly optimized and implemented [39].

### Blood Sampling

Home-based STI and HIV testing requires individuals to take samples at home. This may be challenging, especially for blood self-collection (regarding adequate volumes capillary) [10, 18, 32]. For syphilis and HIV laboratory testing on blood, a certain minimum of blood volume is required for valid diagnostics [10, 18, 32]. Sampling blood at home is usually done via finger-prick blood collection, though in recent years alternative methods have been developed. An example of innovative blood collection methodology is the use of a semi-automatic vacuum microneedle [31, 49]. The assessed at-home device (Tasso+) allowed the user to semi-automatically collect blood from the upper arm through a vacuum mechanism. This blood collection procedure was found highly acceptable and feasible for collecting larger volumes of blood enabling testing and including confirmation testing for various STIs including HIV. A US study showed promising results in testing such device on a larger scale, along with real-world logistic possibilities [50].

A similar upper arm blood self-collection device, the TAP II device, has only been tested among patients with immune-mediated inflammatory rheumatic disease. In this study the upper-arm collection methods were usable, and half of the patients preferred the device compared to venous blood collection (by an health care provider) [51]. Another similar device (Onflow) reportedly provided painless blood collection from the upper arm. However, to date, this device has only been assessed under the supervision of health care providers, who were able to correctly apply the device [52]. A potential limitation of these upper arm methods is their higher cost compared to finger-prick blood collection; however, no studies to date have evaluated this issue.

### Digital Communication and Services Provision Methods

In a pre-implementation study among MSM, their preferences in types of interactions with an health care provider in future home-based sexual health care service were evaluated [26]. MSM reported a preference for telephone and webcam contact with an health care provider [36], aligning with findings among PrEP using MSM [31].

Rather than contact with a ‘real person’, contact with a chatbot is increasingly used in sexual health care. An example of applying a chatbot in sexual health care is a pilot study among MSM in Malaysia which determined interaction with AI chatbots to be facilitating in accessing HIV testing and sexual health information [53]. In the Netherlands, an online chatbot (Advies.chat) is available to advise on cues to action that the individual should take based on answers to questions such as about specific symptoms [54]. In a recent narrative review, the advantages and challenges of chatbots for HIV prevention were assessed [55]. For individuals who experienced challenges in contacting an health care provider, engaging with a chatbot may be a solution. It may be experienced as easy, not stigmatizing for those who seek sexual health care (information), potentially leading to the uptake of HIV testing. However, applying chatbots in sexual health care in counseling may raise concerns considering the safety and ethicality of data, as well as the correctness of medical advice given by chatbots when using AI algorithms.

Another useful tool for accessing home-based sexual health care may be mobile applications (LYNX and MyChoice), where people can order their test and see their results via an app, as evaluated among young MSM in the US [56]. In a qualitative study among young MSM from China the feasibility of app-based health care was confirmed [57]. Here again, confidentiality concerns regarding information security were expressed. Especially younger individuals may benefit from digital communication and services provision since they obtain most of their sexual health information through digital sources (i.e., internet, and mobile applications) [58].

In previous research, online communication for the distribution of home-based sexual health care was the preferred communication mode [26], but offline communication (i.e., posters, cards, and word-of-mouth communication) should be available for populations that have insufficient access to digital technology or otherwise have low (digital) health literacy [25, 59]. “Limburg4zero” employed a broad and tailored communication strategy that used a mixed approach of online and offline channels to reach out to diverse groups of MSM [18]. Social media could be a mode to disseminate the care service. In-depth interviews with MSM from the US demonstrated a positive attitude toward advertisements about PrEP in dating apps and participants deemed the advertisements to be effective [60]. A strategy for online dissemination is by targeting. A recent case study (Open door Health) demonstrated the effectiveness of targeted search engine advertisements for increased use of STI and HIV testing services [61].

Recently the WHO released a technical briefing on using AI in sexual health, stating this as a facilitator of access to sexual health care [62]. A review study from the US identified possible AI applications in preventing HIV. A key finding was the potential of AI in the early identification of individuals who were at high risk for HIV exposure [63]. An Australian study evaluated an AI tool (*MySTIRisk*) that used machine learning to identify patients at elevated risk for STI and HIV from a public health center database [64]. The machine learning tool was successful in determining the priority populations, who were at risk for HIV exposure. Consequently, identified priority populations could be offered early testing and preventive options including PrEP.

Another AI development is to aid clinical diagnosis. For instance, the identification of STI by using images of the skin (HeHealth) [65]. Particularly for STI such as syphilis and mpox, this presents an opportunity for individuals to photograph their symptoms and perform self-assessments at home. Still, ethical questions are also raised about the privacy and security of data concerning a person’s sexual health [44]. The WHO called for AI applications as facilitators for accessing sexual health care while ensuring human rights and patients’ privacy [62].

## Conclusion

Home-based sexual health overall increases the accessibility to sexual health care, STI, and HIV testing for previously untested MSM. It also offers an alternative to engage MSM that have attended location-based care, such as for PrEP. Home-based care can be a stand-alone service yet is also strong as a more comprehensive care offer that is offered as a complementary service integrated within location-based sexual health care. Implementing home-based strategies ultimately strives for achieving improved sexual health by an equitable healthcare offer. Nevertheless, ensuring equity is a continuous challenge not solved by the home-based services currently in place. Future research needs to specifically address accessibility of sexual health care for vulnerable individuals, still underserved by current care options. Therefore, low-threshold location-based care should remain an alternative, and developing secure digital sexual health solutions should be encouraged, specifically addressing priority populations’ health care needs. Continuous evaluation of (usage of) the home-based care-elements as well as the implementation of the care should be pursued where possible, to optimize home-based care and its accessibility and the seamless integration of this service into routine sexual health care.

### Recommendations for Practice Implications

Our recommendations regarding the integration, implementation, and development of home-based sexual health care are as follows:

- **Implement home-based sexual health care complementary to existing location-based sexual health care.** Home-based sexual health care is highly acceptable for priority populations, and it should be integrated into existing location-based sexual health care. Doing so combines the advantages of both care forms such as supporting the self-autonomy of its users (since they may choose where and when they want to utilize sexual health care services) and health care providers can ensure the quality of care when the home-based care is offered by this highly skilled health care provider, who can also link the patient to location-based care when necessary.
- **Tailoring of the care offer and its implementation is essential for improving the accessibility of home-based sexual health care.** To address the preferences of the priority population and subgroups, it is necessary to tailor the home-based service to enhance its accessibility. For instance, by performing a needs assessment before implementation, a home-based sexual health care service may be customized to fit the preferences of the priority population. A systematic optimization of the intervention itself, for example by using the systematic Intervention Mapping framework [66], and of its implementation strategies for maximal promotion of its use in various priority groups, is recommended.
- **Follow an implementation science framework.** To systematically implement a care intervention, specifically a home-based sexual health care service, we recommend adherence to an implementation science framework. The use of a systematic implementation framework to provide a certain level of detail and tailoring of the implementation strategies to optimally harness the benefits of the home-based care intervention in practice, is beneficial to use by health care providers and scientists, learn from, and work sequentially to progress the field. A systematic approach can facilitate a successful and structured implementation process, eventually leading to the desired outcome which is improved sexual health for all.

## Key References

- Flowers P, Riddell J, Park C, Ahmed B, Young I, Frankis J, et al. Preparedness for use of the rapid result HIV self-test by gay men and other men who have sex with men (MSM): a mixed methods exploratory study among MSM and those involved in HIV prevention and care. HIV Med [Internet]. 2017;18:245–55. Available from: https://onlinelibrary.wiley.com/doi/10.1111/hiv.12420.
-
  - This study shows the acceptability of home-based sexual health care services among health care providers.
- Goense CJD, Doan THP, Kpokiri EE, Evers YJ, Estcourt CS, Crutzen R, et al. Understanding Practical, Robust Implementation and Sustainability of Home-based Comprehensive Sexual Health Care: A Realist Review. AIDS Behav. 2024.
-
  - This realist review helps understand key elements that enhance the implementation of home-based sexual health care among priority populations.
- Rapid Response Service. A review of internet-based testing services for HIV and sexually transmitted infections (STIs) [Internet]. Toronto, ON; 2022 Mar. Available from: https://www.ohtn.on.ca/rapid-response-a-review-of-internet-based-testing-services-for-hiv-and-sexually-transmitted-infections-stis/
-
  - This source summarizes current, promising home-based testing services for STI.
- Goense CJD, Evers YJ, Manait J, Hoebe CJ, van Loo IH, Posthouwer D, et al. Evaluating the Implementation of Home-based Sexual Health Care among Men Who Have Sex with Men: Limburg4zero. AIDSBehav 2025;
-
  - This study evaluates the implementation process of a regional home-based sexual health care service in the Netherlands.
- Feldstein AC, Russell Glasgow ME. A Practical, Robust Implementation and Sustainability Model (PRISM) for Integrating Research Findings into Practice Research Methods. The Joint Commission Journal on Quality and Patient Safety [Internet]. 2008;34:228–43. Available from: http://www.ihi.org/IHI/Topics/Improvement/ImprovementMethods/HowToImprove/.
-
  - This study explains the PRISM framework’s usability for implementing complex health care innovations.
- Cannon C, Holzhauer K, Golden M. Implementation and Evaluation of a Home-Based Pre-Exposure Prophylaxis Monitoring Option: Protocol for a Randomized Controlled Trial. JMIR Res Protoc [Internet]. 2024;13:e56587. Available from: https://www.researchprotocols.org/2024/1/e56587.
-
  - This study describes an RCT protocol for testing an innovative health care innovation considering self-blood collection in a real-life larger-scale setting.

## Data Availability

No datasets were generated or analysed during the current study.
